# Efficacy and safety of ragweed sublingual immunotherapy in Canadian patients with allergic rhinoconjunctivitis

**DOI:** 10.1186/1710-1492-10-55

**Published:** 2014-11-10

**Authors:** Harold Kim, Susan Waserman, Jacques Hébert, Michael Blaiss, Harold Nelson, Peter Creticos, Amarjot Kaur, Jennifer Maloney, Ziliang Li, Hendrik Nolte

**Affiliations:** McMaster University, Hamilton, ON Canada; Western University, London, ON Canada; Centre de Recherche Appliquée en Allergie de Québec, Québec, QC Canada; University of Tennessee Health Science Center, Memphis, TN USA; National Jewish Health, Denver, CO USA; Johns Hopkins University School of Medicine, Baltimore, MD USA; Creticos Research Group, Crownsville, MD USA; Merck & Co., Inc., Whitehouse Station, NJ USA; 525 Belmont Ave West, Suite 205, Kitchener, ON N2M 5E2 Canada

**Keywords:** Allergic rhinitis, Conjunctivitis, Ragweed pollen, Allergen immunotherapy, Sublingual immunotherapy Tablet, SLIT

## Abstract

**Background:**

Currently accepted therapies for ragweed allergy in North America consist of pharmacotherapy and subcutaneous allergen immunotherapy injections to treat symptoms. Allergen immunotherapy not only reduces symptoms and the need for pharmacotherapy but has also been shown to have disease-modifying potential. Recently, ragweed immunotherapy administered via sublingual allergen tablet has been approved in North America for treatment of allergic rhinitis with and without conjunctivitis.

**Methods:**

This was an analysis of pooled data for a prespecified subgroup of Canadian subjects from two multicentre, randomized, double-blind placebo-controlled trials of ragweed sublingual tablet (SLIT-T; 6 and 12 Amb a 1-U of *Ambrosia artemisiifolia*) in patients aged ≥18y, with ragweed-induced allergic rhinoconjunctivitis (AR/C) with or without asthma. Randomized subjects used once-daily ragweed SLIT-T or placebo for at least 12 weeks before the ragweed season and for up to 52 weeks post-randomization. The primary efficacy endpoint was the total combined score (TCS) based on the sum of AR/C daily symptom score (DSS) and daily medication score (DMS) averaged over the peak season. Treatment effects on TCS, DSS, and DMS in the entire season were also assessed. Adverse events (AEs) were monitored to assess safety.

**Results:**

337 Canadian subjects were randomized in the two trials. During the peak season, ragweed SLIT-T 6 and 12 Amb a 1-U significantly reduced TCS by 26% (difference, -2.46 score point; p = .0009) and 40% (difference, -3.75 score point; p < .0001), respectively. In the overall population (N = 961), TCS reductions with 6 and 12 Amb a 1-U were 20% and 23%, respectively (both p < .001). Clinically meaningful reductions in entire-season TCS in Canadians were similar to those during peak ragweed season. Dose-dependent reduction of DSS and DMS was also observed for ragweed SLIT-T 6 and 12 Amb a 1-U during the peak season and the entire season. Ragweed SLIT-T was well tolerated in Canadian subjects and the overall population. Adverse events were generally mild to moderate and transient, occurring early in treatment; no systemic allergic reaction/anaphylaxis was noted.

**Conclusion:**

Ragweed SLIT-T is an effective form of immunotherapy that provides symptomatic efficacy of AR/C with a favorable risk profile in Canadian and overall populations.

**Trial registration:**

Clinicaltrials.gov identifiers NCT00783198 and NCT00770315.

## Introduction

Short or “common” ragweed (*Ambrosia artemisiifolia*) pollen is among the more common causes of respiratory allergies in North America [1]. Ragweed allergy is a longstanding treatment challenge in eastern Canada [2, 3], and the recent lengthening of ragweed pollen season, which is especially pronounced in higher latitudes of North America, has contributed to the problem [4]. Ragweed is common in the provinces of Quebec and Ontario, while it is rare in British Columbia [5]. Results of allergy testing of 2595 individuals in regions across Canada found the overall prevalence of allergen sensitization was 62.7%, and up to 33% of subjects were positive to ragweed [6].

Allergic rhinitis with or without conjunctivitis (AR/C) is typically induced by animal dander, dust mites, moulds and pollens. The first line of treatment is avoidance, but allergen avoidance is difficult with pollens, necessitating the use of pharmacotherapy [7]. Treatment of ragweed allergy with subcutaneous injections has also been shown to be effective [8–10]. Unlike pharmacotherapy, immunotherapy targets the immunologic cause of respiratory symptoms and is the only treatment that alters the course of respiratory allergic disease [7]. However, the inconvenience of injections may make some patients reluctant to undergo this form of treatment. Studies have found that factors related to both inconvenience and safety contribute to premature discontinuation of subcutaneous immunotherapy [11, 12].

The treatment of ragweed allergy with ragweed sublingual immunotherapy tablet (SLIT-T) MK-3641 (12 Amb a 1-U of *Ambrosia artemisiifolia*; Merck & Co, Inc., Whitehouse Station, NJ, USA/ALK-Abelló, Hørsholm, Denmark) has been approved as a new treatment option for patients with AR/C. Two large North American trials found MK-3641 6 and 12 Amb a 1-U to be effective and well tolerated for ragweed-pollen–induced AR/C [13, 14]. These two trials involved Canadian subpopulations, and it was of interest to examine the effects of ragweed SLIT-T in Canadian subjects in view of the significant morbidity caused by ragweed in that population [15].

## Methods

### Study design

An analysis of pooled data for the pre-specified subgroup of Canadian subjects from two multicentre, randomized, double-blind placebo-controlled trials of MK-3641 6 and 12 Amb a 1-U in patients with ragweed-induced AR/C with or without asthma was conducted. One trial (protocol P05233; clinicaltrials.gov identifier NCT00783198) included 13 centres in Canada, and the other trial (protocol P05234; clinicaltrials.gov identifier NCT00770315) included 12 centres in Canada. Both trial protocols were approved by local or centre-specific ethic review boards (P05233, IRB Services; P05234, BMC REB Montreal Chest Institute of the MUHC, Hamilton Health Sciences/Faculty of Health Sciences Research Ethics Board, and IRB Services), and all patients gave written informed consent. Trial P05234 included subjects randomized to a dose of MK-3641 (1.5 Amb a 1-U) that was found clinically ineffective and was excluded from the pooled analysis. Trial P05233 did not assess a dose of 1.5 Amb a 1-U. Both trials were conducted over the 2010 ragweed season and had the same study design; the methodologies have been published elsewhere [13, 14].

The primary hypothesis in both trials was that ragweed SLIT-T is superior to placebo as measured by improvement in total combined score (TCS) averaged during the peak ragweed pollen season (RS). The peak RS was defined as the 15 consecutive recorded days within RS with the highest 15-day moving average pollen count for each site. The RS was defined as the period from the first day of three consecutive recorded days with a pollen count of ≥10 grains/m^3^ to the last day of the last occurrence of three consecutive recorded days with a pollen count ≥10 grains/m^3^, inclusively [13].

Randomized subjects were treated once daily with either MK-3641 or placebo for at least 12 weeks before the RS, during the entire RS, and after the end of RS for up to 52 weeks post-randomization. MK-3641 dissolves within seconds of application, does not require up-titration, and was administered at the office for the first three doses and then self-administered at home for the remainder of the trial. As an added safety measure, all subjects were supplied with self-injectable epinephrine and instructions on how and when to use it. Open-label rescue medications for AR/C were provided. Key inclusion and exclusion criteria are listed below.

### Key eligibility criteria

#### Inclusion criteria

- Patients had to have been willing to give informed written consent and be able to adhere to dose and visit schedules
- 18 to 50 years of age, of either sex and of any race
- Clinical history of significant ragweed-induced AR/C of two years’ duration or more with or without asthma (diagnosed by a physician) and have received treatment for the disease during the previous RS
- Positive skin prick test response to *Ambrosia artemisiifolia* (wheal size ≥5 mm larger than saline control) at the screening visit
- Positive for specific IgE against *Ambrosia artemisiifolia* (≥IgE Class 2 [≥0.70 kU/L]) at the screening visit.
- Forced expiratory volume in 1 second of at least 70% of predicted value at the screening visit
- Safety laboratory tests and vital signs conducted at the screening visit must be within normal limits or clinically acceptable to the investigator/sponsor

#### Exclusion criteria

- Clinical history of symptomatic seasonal allergic rhinitis and/or asthma having received regular medication, owing to another allergen during or potentially overlapping the RS
- Clinical history of significant symptomatic perennial allergic rhinitis and/or asthma having received regular medication due to another allergens to which the subject is regularly exposed
- Received an immunosuppressive treatment within three months prior to the screening visit (except steroids for allergic and asthma symptoms)
- Clinical history of severe asthma
- History of asthma requiring medium- or high-dose inhaled corticosteroids
- History of anaphylaxis with cardiorespiratory symptoms
- History of chronic urticaria and angioedema
- Clinical history of chronic sinusitis during the two years prior to the screening visit
- Current severe atopic dermatitis
- Female subject who was breastfeeding, pregnant, or intending to become pregnant
- Previous immunotherapy treatment with ragweed allergen or any other allergen within the five years prior to the screening visit
- History of allergy, hypersensitivity or intolerance to the ingredients of the medicinal products (except for *Ambrosia artemisiifolia*), rescue medications, or self-injectable epinephrine
- History of self-injectable epinephrine use

AR/C, allergic rhinitis with or without conjunctivitis; IgE, immunoglobulin E; RS, ragweed pollen season.

### Efficacy assessment

The primary efficacy endpoint was the TCS based on the combined (sum of) AR/C daily symptom score (DSS) and daily medication score (DMS) averaged over the peak RS. Symptoms and medication use were recorded in an electronic diary once daily in the evening before bed. A total of six AR/C symptoms (runny nose, blocked nose, sneezing, itchy nose, gritty eyes, and watery eyes) were scored on a scale of 0 (none) to 3 (severe), for a maximum total daily symptom score of 18 [13]. Daily rescue medication use for AR/C was transformed to a DMS by applying score/dose units for desloratadine, olopatadine ophthalmic solution, mometasone furoate nasal spray, and prednisone tablets (Table 1) [13]. Secondary endpoints included TCS over the entire season as well as the individual DSS and DMS averaged over the peak and entire season.

**Table 1:** **Scoring of rescue medication use** [13]

Rescue medication	Score/dose unit	Maximum daily score
Desloratadine (10 mg/tablet) – 1 tablet QD	6/tablet	6
Olopatadine hydrochloride 0.1% ophthalmic solution (one drop in the affected eye BID)	1.5/drop	6
Mometasone furoate nasal spray 50 μg QD (2 sprays in each nostril)	2/spray	8
Prednisone tablet 5 mg (Day 1: 1 mg/kg/d; max 50 mg/d; days 2+: 0.5 mg/kg/d; max 25 mg/d)	1.6/tablet	16
TOTAL		36

### Safety assessment

Adverse events (AEs), vital signs, physical examinations, and safety laboratory assessments were summarized by overall treatment groups.

### Statistical analysis

Analyses were performed based on pooled data across two studies for the entire population as well as the Canadian subpopulation. The efficacy endpoints were analyzed using analysis of variance model adjusted for factors such as treatment, pollen region, and asthma status. The least squares mean and two-sided 95% CI for the between-treatment differences were estimated from the models for the entire population, with the associated p values reported.

Multiplicity control on testing multiple endpoints was applied by using a stepwise method for the entire population analyses, and nominal p values without any adjustment for multiplicity were reported for the Canadian subgroup analyses. The percentage reduction relative to placebo effect was calculated as (MK-3641–placebo)/placebo × 100% using the within-group least squares means for the MK-3641 group and the placebo group.

Patients who took at least one dose of study medication and who had at least one post-randomization diary record were included in the efficacy analysis [13].

## Results

### Baseline demographics

Demographics for the entire population of the two trials have been published previously [13, 14], but are included here because results for Canada are presented in the context of results for the entire tested population. The Canadian subpopulation receiving MK-3641 6 Amb a 1-U, MK-3641 12 Amb a 1-U, or placebo included 337 subjects in the two pooled trials. The treatment groups had similar characteristics at randomization; 89–95% of patients were also sensitized to multiple nonragweed allergens including other pollen and perennial allergens, and 22–27% of patients reported concomitant intermittent-to-mild stable asthma with predicted FEV_1_ > 70% (Table 2). Across all treatment groups in the entire randomized population, 80–82% of patients were polysensitized and 18–21% of patients had concurrent asthma. At randomization, Canadian subjects had mean specific immunoglobulin E (sIgE) against *A. artemisiifolia* at serum levels ranging from 17.8–20.4 kU/L across the treatment groups (Table 2). In contrast, the entire population had mean sIgE (*A. artemisiifolia*) serum levels ranging from 14–18 kU/L across the treatment groups [13, 14].

**Table 2:** **Demographics and baseline characteristics in the Canadian subgroup pooled for two studies**

Characteristic	6 Amb a 1-U MK-3641 (n =112)		12 Amb a 1-U MK-3641 (n =110)		Placebo (n =115)	
**Female, n (%)**	51 (45.5)		59 (53.6)		49 (42.6)	
**Age, mean (range), y**	36.2 (18.0–50.0)		35.8 (19.0–51.0)		36.9 (18.0–51.0)	
**Race, n (%)**						
White	84 (75.0)		93 (84.5)		86 (74.8)	
Black or African American	7(6.3)		6 (5.5)		7 (6.1)	
American Indian or Alaskan native	2 (1.8)		0		0	
Asian	17 (15.2)		9 (8.2)		20 (17.4)	
Multiracial	2 (1.8)		2 (1.8)		2 (1.7)	
**History of asthma, n (%)**	22 (19.6)		23 (20.9)		27 (23.5)	
**Tobacco use, n (%)**	31 (27.7)		24 (21.8)		33 (28.7)	
**Duration of ragweed allergy, mean, y**	18.5		17.4		17.4	
**Skin prick test, mean (SD) wheal measurement, mm**						
Histamine positive control	n = 111	5.7 (1.66)	n = 108	5.7 (1.45)	n = 114	5.6 (1.60)
Saline negative control	n = 111	0.7 (1.20)	n = 108	0.6 (1.04)	n = 114	0.5 (0.99)
*Ambrosia artemisiifolia* extract	n = 111	12.2 (4.48)	n = 110	12.1 (3.96)	n = 115	11.8 (4.03)
**Serum sIgE (** ***Ambrosia artemisiifolia*** **), mean (SD) kU/L**	n = 112	17. 8 (19.8)	n = 110	19.4 (23.4)	n = 115	20.4 (23.2)
**Polysensitized, n (%)**	95 (84.8)		89 (80.9)		90 (78.3)	

Amb a 1-U, *Ambrosia artemisiifolia* units; sIgE, specific immunoglobulin E.

### Ragweed season

The average ragweed season in Canada lasted approximately 31 days and was characterized by mean peak pollen counts of 103 grains/m^3^ in trial P05233 and 95 grains/m^3^ in trial P05234. In the entire population, the average ragweed season lasted approximately 45 days and was characterized by mean peak pollen counts of 122 grains/m^3^ in trial P05233 and 127 grains/m^3^ in trial P05234.

### Efficacy results

**Figure 1:**
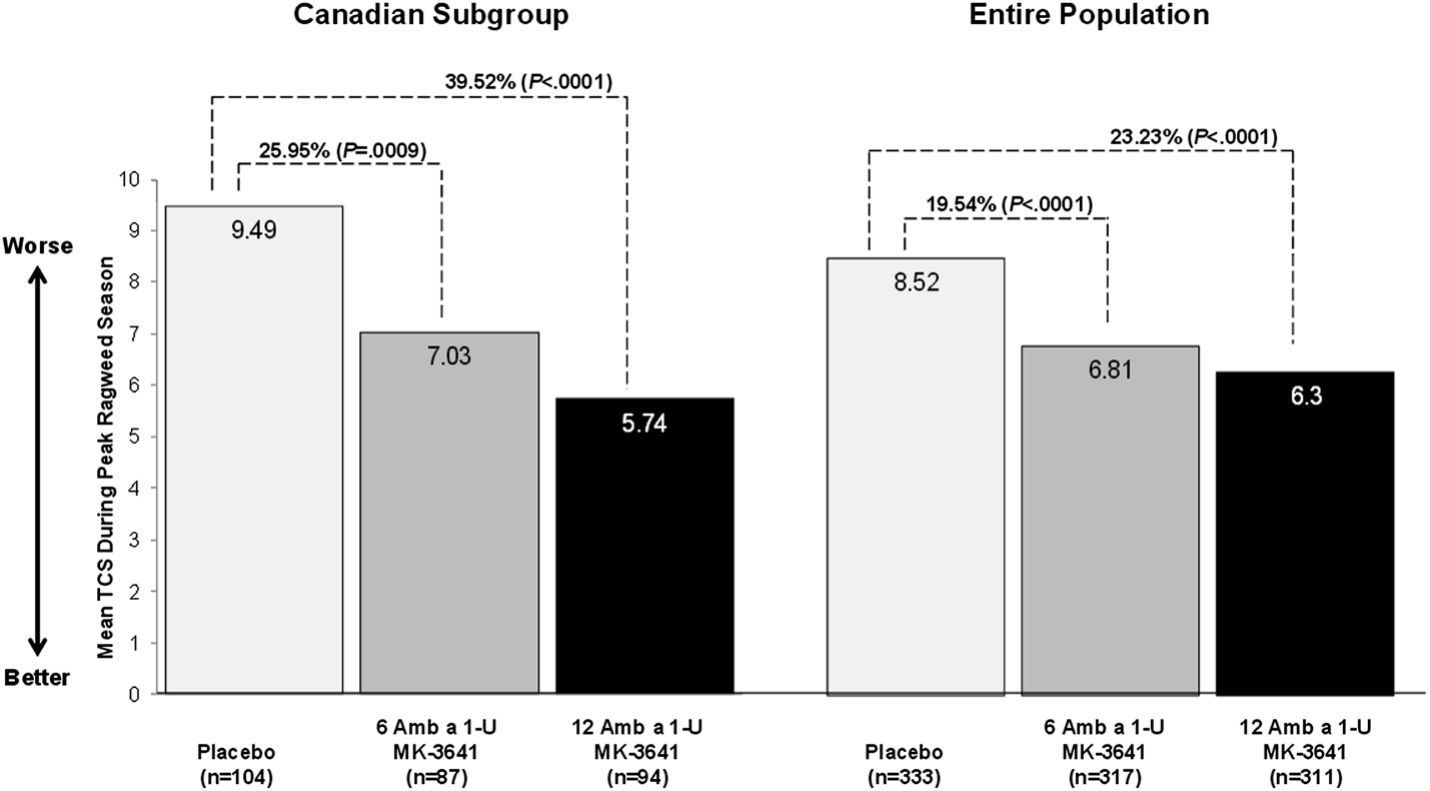
**Pooled results for mean TCS during the peak ragweed pollen season in the Canadian subgroup and the entire population in two trials.**

**Figure 2:**
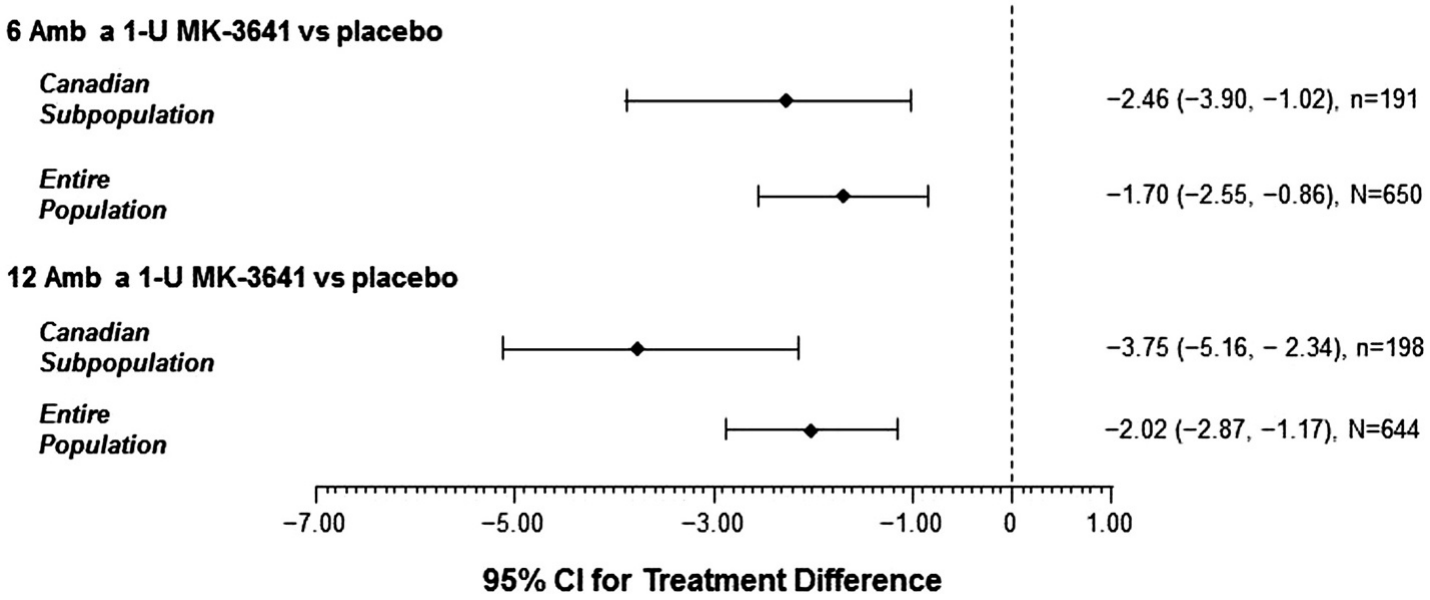
**Differences between mean effects of MK-3641 and placebo on TCS during peak ragweed pollen season in the Canadian subgroup and the entire population in two trials.**

The overall treatment effect of MK-3641 was dose dependent. Compared with placebo during the peak season, MK-3641 6 and 12 Amb a 1-U significantly reduced TCS among Canadian subjects by 26% (difference, -2.46 score point; p = .0009) and 40% (difference, -3.75 score point; p < .0001), respectively, in the pooled data for the two trials, and these results were generally consistent with those seen in the overall North American study population (Figure 1). Mean TCS differences from placebo during the peak season ranged from -1.70 to -3.75 overall, with a trend toward slightly greater reductions in the Canadian subgroup than in the entire population (Figure 2). During the entire season, the pooled data revealed that MK-3641 6 and 12 Amb a 1-U significantly reduced TCS among Canadian subjects by 22% (difference, -1.75 score point; p = .0036) and 37% (difference, -2.91 score point; p < .0001), respectively, compared with placebo (Table 3).

**Table 3:** **Mean TCS, DSS, and DMS during the peak season and the entire season in the Canadian subgroup**

	Peak season			Entire season		
	6 Amb a 1-U MK-3641 (n = 87)	12 Amb a 1-U MK-3641 (n = 94)	Placebo (n = 104)	6 Amb a 1-U MK-3641 (n = 88)	12 Amb a 1-U MK-3641 (n = 94)	Placebo (n = 104)
**TCS**	**7.03**	**5.74**	**9.49**	**6.06**	**4.91**	**7.82**
Difference vs placebo (95% CI)	-2.46 (-3.90, -1.02)	-3.75 (-5.16, -2.34)		-1.75 (-2.93, -0.58)	-2.91 (-4.07, -1.75)	
p value	.0009	< .0001		.0036	< .0001	
Reduction vs placebo	25.95%	39.52%		22.44%	37.23%	
**DSS**	**5.09**	**4.44**	**5.95**	**4.51**	**3.91**	**5.05**
Difference vs placebo (95% CI)	-.86 (-1.78, .06)	-1.52 (-2.42, -.61)		-.54 (-1.33, .24)	-1.14 (-1.92, -.37)	
p value	.0677	.0011		.1746	.0038	
Reduction vs placebo	14.46%	25.49%		10.72%	22.66%	
**DMS**	**1.93**	**1.30**	**3.53**	**1.55**	**1.00**	**2.77**
Difference vs placebo (95% CI)	-1.60 (-2.36, -.84)	-2.23 (-2.97, -1.49)		-1.21 (-1.81, -.61)	-1.77 (-2.35, -1.18)	
p value	< .0001	< .0001		< .0001	< .0001	
Reduction vs placebo	45.29%	63.14%		43.82%	63.84%	

Amb a 1-U, *Ambrosia artemisiifolia* units.

In the Canadian subgroup, dose-dependent reductions of mean DSS and DMS were also observed for MK-3641 6 and 12 Amb a 1-U during the peak ragweed season as well as the entire season. Reductions of peak-season scores, when symptoms and medication use are at their highest levels, were generally greater than reductions during the entire season (Table 3). Overall, during the peak and entire ragweed seasons, reduction of DSS was statistically significant for MK-3641 12 Amb a 1-U vs placebo (p ≤ .0038) but not for MK-3641 6 Amb a 1-U vs placebo (p ≥ .0677). During the peak season, DSS reductions of 14% and 25%, respectively, occurred with MK-3641 6 and 12 Amb a 1-U compared with placebo. Reductions in DSS of 11% and 23%, respectively, occurred with MK-3641 6 and 12 Amb a 1-U during the entire season. During the peak season, DMS reductions of 45% and 63%, respectively, occurred with MK-3641 6 and 12 Amb a 1-U compared with placebo. Reductions in DMS of 44% and 64%, respectively, occurred with MK-3641 6 and 12 Amb a 1-U during the entire season. During the peak and entire seasons, DMS reductions were statistically significant for both doses of MK-3641 vs placebo (p < .0001).

### Safety results

MK-3641 was well tolerated in the Canadian subgroup as well as the entire study population. Adverse events were generally assessed as mild or moderate and transient, and they generally occurred early in treatment. No deaths, treatment-related systemic allergic reactions, or serious treatment-related serious AEs occurred. Local reactions of the mouth, throat, and ear were the most common treatment-related AEs (TRAEs) (Table 4) [13, 14]. In the Canadian subpopulation, 34%, 75%, and 81% of subjects in the placebo, 6 Amb a 1-U, and 12 Amb a 1-U groups, respectively, experienced a TRAE. In the entire population, 25%, 55%, and 61% of subjects in the placebo, 6 Amb a 1-U, and 12 Amb a 1-U groups, respectively, experienced a TRAE. Overall, there was a non-significant trend toward more AEs and a higher discontinuation rate due to AEs in the Canadians compared to the entire population. A 21-year-old woman who received MK-3641 6 Amb a 1-U was administered epinephrine in an emergency department in response to mild pharyngeal edema and other local allergic events. The subject had experienced local events during prior administrations of the tablet. The investigator considered the AE probably treatment-related, although the differential diagnosis on admission to the emergency department was allergic reaction versus anxiety. The subject recovered from the event and discontinued MK-3641.

**Table 4:** **Summary of overall adverse events in the entire population and the Canadian subpopulation in two trials**

Entire population	Entire population			Canadian subpopulation		
	6 Amb a 1-U MK-3641 (n =385)	12 Amb a 1-U MK-3641 (n =381)	PBO (n =386)	6 Amb a 1-U MK-3641 (n =112)	12 Amb a 1-U MK-3641 (n =110)	PBO (n =115)
**AE Category, n (%)**						
Any TEAEs	296 (76.9)	307 (80.6)	264 (68.4)	100 (89.3)	104 (94.5)	93 (80.9)
Any TRAEs	213 (55.3)	233 (61.2)	98 (25.4)	84 (75.0)	89 (80.9)	39 (33.9)
Any serious TEAEs	5 (1.3)	3 (0.8)	4 (1.0)	1 (0.9)	0	1 (0.9)
Any serious TRAEs	0	0	0	0	0	0
Any TEAEs leading to study discontinuation	31 (8.1)	35 (9.2)	9 (2.3)	16 (14.3)	15 (13.6)	2 (1.7)
Any TRAEs leading to study discontinuation	26 (6.8)	31 (8.1)	6 (1.6)	14 (12.5)	14 (12.7)	1 (0.9)
**TRAEs in ≥5% of subjects, n (%)**						
Oral pruritus	83 (21.6)	66 (17.3)	10 (2.6)	40 (35.7)	36 (32.7)	7 (6.1)
Ear pruritus	54 (14.0)	54 (14.2)	6 (1.6)	24 (21.4)	30 (27.3)	2 (1.7)
Throat irritation	90 (23.4)	96 (25.2)	21 (5.4)	39 (34.8)	43 (39.1)	9 (7.8)
Mouth edema	38 (9.9)	37 (9.7)	2 (0.5)	20 (17.9)	17 (15.5)	2 (1.7)
Eye pruritus	16 (4.2)	15 (3.9)	5 (1.3)	9 (8.0)	5 (4.5)	1 (0.9)
Nasal passage irritation	14 (3.6)	15 (3.9)	14 (3.6)	7 (6.3)	5 (4.5)	2 (1.7)
Skin pruritus	24 (6.2)	10 (2.6)	6 (1.6)	12 (10.7)	3 (2.7)	2 (1.7)

AE, adverse event; PBO, placebo; TEAE, treatment-emergent adverse event; TRAE, treatment-related adverse event.

## Discussion

In the two efficacy trials evaluating MK-3641, a significant reduction in symptoms and medication usage versus placebo among the Canadian subjects with ragweed-pollen–induced AR/C was demonstrated. These trials illustrated a dose–response effect of once-daily MK-3641, with 12 Amb a 1-U showing the greatest reductions in the primary endpoint of TCS during the peak RS. Treatment with 12 Amb a 1-U ragweed allergy immunotherapy tablet provided significant clinical benefit to AR/C subjects, as shown by improvement in the TCS, DSS, and DMS during the peak RS, when subjects were suffering the most due to the highest exposure to ragweed pollen. In addition to providing clinical effects during the height of the pollen season, similar findings occurred during the entire ragweed pollen season. Furthermore, results in Canadian subjects were similar to results observed in the entire North American study population.

Overall safety profiles of MK-3641 6 and 12 Amb a 1-U were similar, with a numerically greater treatment effect for 12 Amb a 1-U. Hence, the recommended therapeutic dose is 12 Amb a 1-U. The first three days of treatment with MK-3641 were administered in the physician’s office, although trials with sublingual grass immunotherapy tablet have shown that one day of in-office treatment is sufficient and three days of in-office treatment does not provide an additional safety advantage [16]. Treatment with MK-3641 was well tolerated for up to one year. The most frequently reported TRAEs occurred early and were transient, self-limited local reactions of the mouth, throat, and ears (primarily oral pruritus, throat irritation, and ear pruritus), and the majority of events were assessed as mild or moderate in severity. No serious TRAEs or systemic reactions were reported, and no upper airway obstruction, death, anaphylactic shock or life-threatening events occurred. However, there was a trend toward more adverse events in the Canadian subgroup, the significance of which is uncertain considering that the study was not designed or powered for this assessment. There was also a trend toward greater efficacy in the Canadian subgroup. These trials assessed one year of treatment for ragweed AR/C, while immunotherapy typically is maintained for several years. Also, it has been suggested that local application site reactions may compromise blinding of treatments and bias the results [17]. A predefined exploratory assessment of treatment with MK-3641 12 Amb a 1-U and placebo in subjects with or without local reactions suggests unblinding due to local reactions did not impact the overall efficacy assessment [18].

Guidelines from the World Allergy Organization and Allergic Rhinitis and its Impact on Asthma emphasize that high-dose sublingual immunotherapy is effective, well tolerated, and safe for self-administration [19, 20]. Previously, only a limited number of studies have evaluated sublingual treatments for ragweed allergy [21–24]. Two of these trials were conducted in Canada [22, 24]. The trials of MK-3641 differed from the previously published Canadian ragweed trials in several important ways. First, MK-3641 is a sublingual immunotherapy tablet, whereas the other trials evaluated a liquid sublingual swallow extract [22, 24]. MK-3641 was administered to randomized subjects at a fixed dose of 6 or 12 Amb a 1-U from the beginning to the end of treatment, without up-titration. By contrast, the sublingual swallow extracts were administered with an initial progression of increasing doses, and the highest-tolerated dose was used for maintenance treatment [22, 24]. One of the sublingual swallow trials evaluated ragweed extract doses of at least 116 Amb a 1-U, with a target dose of 348 Amb a 1-U [22]. The other sublingual swallow trial evaluated extract doses of up to 50 Amb a 1-U [24], whereas the present MK-3641 trials evaluated maximum doses of 12 Amb a 1-U. Results from one sublingual swallow extract trial showed no significant effect on the total rhinitis, conjunctivitis, or medication scores [22], whereas the other showed significant reduction of total combined and daily symptoms scores but did not evaluate medication scores [24]. These trials used higher doses of Amb a 1-U than were used in the MK-3641 trial. Conversely, MK-3641 treatment resulted in significant improvements of daily symptom and medication scores as well as the total combined score.

## Conclusions

MK-3641, a novel therapeutic modality of ragweed SLIT-T, significantly improved AR/C caused by ragweed pollen in Canadian adults aged ≥18 years. MK-3641 is an effective form of immunotherapy that provides symptomatic efficacy of AR/C with a favorable risk profile. Local allergic events are expected with sublingual administration of allergen. Although these events occur, they are manageable, resolve typically without treatment, and have not resulted in airway obstruction. No concerning or new safety signals emerged, and MK-3641 was tolerated in subjects with intermittent-to-mild stable asthma. In conclusion, these trials support the use of 12 Amb a 1-U ragweed SLIT-T for self-administration at home after the first dose given under medical supervision has been shown to be tolerated. Ragweed sublingual immunotherapy may prove to be a valuable treatment option for patients with ragweed allergy, as suggested by international guidelines.

## Authors’ information

Dr. Blaiss was employed by the University of Tennessee when the studies took place, but is now employed by Merck & Co., Inc., North Wales, PA.
